# Autism spectrum disorder (ASD) presentations among referrals to a child and adolescent mental health service (CAMHS) inpatient unit in Ireland

**DOI:** 10.1177/13591045241295419

**Published:** 2024-10-25

**Authors:** Molly O’Connor, Clair Griffin, Jennifer Corrigan, Carol Somers, Maura Delaney, Fionnuala Larkin

**Affiliations:** 1 School of Applied Psychology, 8795 University College Cork, Co. Cork, Ireland

**Keywords:** Autism spectrum disorder (ASD), retrospective studies, inpatients, child and adolescent, mental health service (CAMHS), comorbidity, child psychiatry

## Abstract

This retrospective chart review aimed to identify the intersection between young people with Autism Spectrum Disorder (ASD)’s needs and CAMHS inpatient service needs. A retrospective chart review was conducted on all referrals to a CAMHS inpatient unit over three years (*n* = 352). Referrals which specified a formal diagnosis or suspected diagnosis of ASD were identified (*n* = 111), and basic demographic data were collected. Young people with either a formal or suspected diagnosis of ASD referred to the unit presented with a wide range of co-occurring conditions. Of these young people, 30 were admitted to the unit and only 6 of them were engaged with an ASD specialist service. Young people with diagnosed or suspected ASD were more likely to be admitted if they presented with suicidality. Males with diagnosed or suspected ASD were more likely to have a diagnosis of OCD while females with diagnosed or suspected ASD were more likely to present with eating and feeding disorders and personality development difficulties. Findings highlight the lack of a clear care pathway for young people with co-occurring autism or suspected autism and complex mental health problems.

## Plain language summary

Many children and adolescents who are referred to Child and Adolescent Mental Health Services (CAMHS) inpatient units may also have autism spectrum disorder (ASD). It is important that we understand their needs and how best services can support them. Methods: In this study, we reviewed the files of all young people referred to a CAMHS inpatient service over a 3 year period (*n* = 352), and identified those where the young person had a diagnosis of ASD, or where the referring clinician suspected the young person may have had autism (*n* = 111). We collected details about their demographics, the mental health difficulties with which they presented, and the services that were supporting them. Results: We found that young people with either a formal or suspected diagnosis of ASD referred to the unit presented with a wide range of co-occurring conditions. Of these young people, 30 were admitted to the unit and only 6 of them were engaged with an ASD specialist service. Young people with diagnosed or suspected ASD were more likely to be admitted if they presented with suicidality. Males with diagnosed or suspected ASD were more likely to have a diagnosis of OCD while females with diagnosed or suspected ASD were more likely to present with eating and feeding disorders and personality development difficulties. Conclusion: Presentations of confirmed and suspected ASD represented a significant number of referrals for inpatient care. This means that it is important for mental health services to work alongside disability services to ensure that the needs of these young people are met.

## Introduction

The intersection between clinical presentations of Autism Spectrum Disorder (ASD) and referrals to inpatient Child and Adolescent Mental Health Services (CAMHS) has received little research attention within an Irish context. ASD is a neurodevelopmental condition, often characterised by differences in social interaction and communication, along with restrictive, repetitive patterns of behaviour or interests (American Psychological Association, 2013). A report published by the Department of Health (2018) stated that the prevalence of ASD in Ireland is estimated to be 1% of the population.

Young people (YP) with ASD can present with acute and complex mental health needs. Over 70% of children with ASD meet the diagnostic criteria for at least one co-occurring psychiatric condition, which frequently persist from childhood to adolescence (Leyfer et al., 2006; Simonoff et al., 2008; Joshi et al., 2010; Simonoff, Jones, Baird, Pickles, & Charman, 2013). Little is known about the relationship between age of ASD diagnosis and comorbid psychopathology (Leader et al., 2022), however, the result of a missed or late diagnosis of ASD can be a gradual cascade of difficulties which permeate various areas of the young person’s life, ultimately precipitating mental health crises requiring specialist inpatient care in some cases (Tchanturia, 2022; Windfall et al., 2022; Lai, Chang, & Skuse, 2022). Croen et al. (2006) reported that children with ASD are six times more likely to be psychiatrically hospitalised than children without ASD. YP with ASD and a co-occurring mental health condition require more care coordination and utilise mental health services more frequently than children with ASD alone (Ahmendani & Hock, 2012). However, service provision for mental health needs secondary to autism can be problematic.

Within the Irish context, the Progressing Disability Services for Children and Young People Programme (PDS) was developed in 2012 to offer access to services for young people with disabilities and/or an ASD profile, including autism assessments (Inclusion Ireland, 2022). Separately, secondary care CAMHS services provide assessment and intervention for YP with moderate to severe mental health difficulties and tertiary care inpatient CAMHS for YP with severe and complex mental health difficulties (Health Service Executive, (HSE), 2019). In effect, young people with autism with complex mental health needs may ‘fall between two stools’, as disability services (Community Disability Network Teams (CDNT’s) will not have the resource to meet severe mental health needs, and mental health services will not have the resource to comprehensively address needs related to autism (e.g. autism assessments). The HSE recommend that disability services, CAMHS, and primary care services engage in joint working to meet patients’ needs (HSE, 2017). However, a report by the Mental Health Commission (2018) noted that the absence of adequate services for children and young people with ASD meant that they are often referred to generic CAMHS services, even if such a referral is inappropriate for their presenting needs. Such referrals are not necessarily accepted by CAMHS services, in line with the CAMHS standard operating procedures and the clinical determination of the triaging CAMHS team. In inpatient services, referrals of YP with an ASD are not accepted in the absence of a moderate to severe mental health disorder (Health Service Executive, 2019). It is vital to quantify the rates of referrals of YP with autism to tertiary services in order to understand the extent of this issue.

Additionally, a quandary arises for clinicians in inpatient settings when a YP referred in crisis for inpatient care may present with a suspected but unconfirmed diagnosis of an autism spectrum condition, which may be due to a missed diagnosis, diagnostic overshadowing, or because the YP is awaiting an autism assessment by the relevant services. Crises prompting referral are therefore often emergent in the context of cumulative stressors secondary to ASD rather than a primary psychiatric condition. Although such a profile might render a referral unsuitable for CAMHS, considerable assessment is often required by triaging clinicians to make this determination. Therefore, the presence or suspicion of ASD has significant implications for care planning by inpatient clinicians, and clarifying the rates of this type of referral is highly important for service provision planning. Furthermore, identification of potential ASD by referring teams is significant as it enables the receiving team to intervene in an autism-informed manner and plan for an appropriate assessment and intervention pathway or for onward referral. Despite this, there is little literature on the issue of undiagnosed ASD in CAMHS in Ireland and the UK. Setlhare and Rafi (2022) examined the profile of YP with undiagnosed ASD in a UK community CAMHS setting, indicating that undiagnosed ASD presents with a wide variety of symptoms, with the most common ages of presentation being 15 and 17.

To our knowledge, no previous research exists within an Irish setting examining the number of young people with an autism profile referred to inpatient CAMHS services. While Rafferty (2021) explored the overall referral and admission patterns to an Irish CAMHS inpatient unit, specific quantification of the referral of young people with an autism profile to inpatient CAMHS has not been completed in an Irish setting. Referrals to CAMHS inpatient services are typically initiated by CAMHS Liaison Psychiatry and Community CAMHS Consultant Psychiatrists, in the event of a severe or emergent mental health crisis, therefore Emergency Departments (EDs) are primary conduits for referrals to CAMHS inpatient care. Internationally, research pertaining to emergent psychiatric presentations suggest that YP with ASD are at a higher likelihood of frequent attendance to EDs in the context of complex psychiatric co-morbidities (Liu et al., 2019; Beverly, Giannouchos, & Callaghan, 2021). Orji and Sharkey (2021) examined presentations of YP with ASD to Child Psychiatry via an Irish ED over a 6-year period, with the authors identifying that a number of repeat presentations to the ED were attributable to disability related factors, with YP on waiting lists for relevant intervention for several years. This speaks to the issue of the complex intersection between autism-related needs and related or consequent mental health needs. Gaining a clearer understanding of whether YP with confirmed/suspected autism attending tertiary services are linked with disability services is therefore another priority.

This retrospective chart review aimed to identify the prevalence of YP with an autism profile across referrals to a CAMHS inpatient unit in Ireland over a three-year period. For the reasons discussed above, referrals with both a confirmed and suspected diagnosis of ASD were examined. The study examined the period 2018–2021 which coincided with the duration of the lockdown phases of the COVID-19 pandemic in Ireland, although the clinical implications of the pandemic are not a focus of this study. The study took an exploratory approach to identify the clinical profile of YP with autism by recording information on basic demographics, co-occurring conditions, and whether young people with confirmed/suspected ASD admitted to the inpatient unit were concurrently engaged with ASD-specific services. This study was exploratory and was guided by the following research questions:

(1) What is the prevalence of ASD (diagnosed or suspected) across referrals and admissions to the inpatient unit over the past 3 years?
(2) What is the demographic and clinical profile of YP with diagnosed or suspected ASD referred to the service?
(3) Are young people with diagnosed ASD who are admitted to the inpatient unit concurrently engaged with ASD-specific services?

In so doing, the study aimed to clarify the level of need of YP with co-occurring autism or suspected autism and complex mental health needs, in order to highlight implications for those providing and resourcing clinical services.

## Method

### Study design

A retrospective chart review was conducted on referrals to a CAMHS inpatient unit over three years (1st January 2018 – 1st January 2021).

### Participants

YP referred to the inpatient unit between 1st January 2018 – 1st January 2021 were included in this study (*N* = 352). Admission criteria for the inpatient unit includes young people ≥12 years of age, who present with a severe or complex mental disorder requiring the level of treatment provided in an inpatient setting, and where the level of risk due to the mental disorder is more appropriately managed in an inpatient setting. On rare occasions where YP were referred to the unit more than once over the three-year period, each referral was treated separately. No exclusion criteria were applied.

#### Data collection

All referral forms made to the inpatient unit during the specified time period were reviewed. The standard referral forms contained free text written by the referrer, typically a consultant psychiatrist, which included demographic information for the young person, reasons for referral, and the doctor’s clinical judgement of the YP’s presenting psychiatric condition or conditions based on their assessment. The primary researcher reviewed all referral forms and collected basic demographic data from them (age, gender). In addition, the presenting psychiatric condition/s were coded by the lead researcher in line with DSM-5 categorisations (APA, 2013). For example, a referral form might state that the YP has difficulties with anxiety and obsessive-compulsive symptoms. This was coded as ‘anxiety disorder’ and ‘obsessive-compulsive disorder’. These were not meant to represent official diagnoses of those YP, but to categorise the main presenting issues in a meaningful way. If the referral form stated that the YP experienced suicidality and deliberate self-harm (DSH), this information was also extracted by the primary researcher.

Referrals which mentioned autism were reviewed and coded as either specifying (1) a diagnosed ASD, or (2) suspected ASD (e.g., discussion of suspected ASD, ‘autistic traits’, neurodiversity or social communication differences). As is standard practice in triage of referrals to the unit, diagnoses were not independently verified, but were based solely on the clinical collateral provided by the referring doctor. For individuals with diagnosed or suspected ASD who were admitted to the unit during the relevant time period, initial referral information was retrieved from case files by the primary researcher. The case files of individuals admitted to the unit were then reviewed to identify whether they were engaged with an ASD-specific service (public or private) at the time of admission.

Missing data (such as not specifying gender, age, or precise referral date) was coded as unknown. All data were manually inputted into Microsoft Excel Version 16.54.

### Data analysis

IBM SPSS statistics version 28 was used to carry out statistical analysis on all data. The data were summarized using descriptive statistics (means, standard deviations for continuous variables such as age and percentages for categorical variables such as gender, ASD diagnosis, co-occurring conditions, admission status). Exploratory inferential statistics were then used to investigate potential associations between variables. While expected cell frequencies were greater than five for most variables, the assumption of chi square was violated for co-occurring conditions with a small sample size (e.g., bipolar and related disorders). In those cases, Fisher’s Exact tests were conducted to explore whether there were statistically significant associations.

## Results

### Referrals and admissions

Three hundred and fifty-two referrals were made to the CAMHS inpatient unit over a three-year period, and 106 admissions were made (Table 1). Referrals with diagnosed or suspected ASD constituted 31.5% (*n* = 111) of all referrals to the unit over the three years. Of 111 referrals indicating ASD, 44.1% (*n* = 49) had a formal ASD diagnosis while 55.9% (*n* = 62) had a suspected diagnosis only. Assuming a 1% prevalence rate of autism in the general population, this means there is a 14-fold chance of being referred for inpatient treatment for YP with a diagnosis of autism.

**Table 1.** Referrals over three years (*n* = 352).

	2018 (*n* = 171)		2019 (*n* = 106)		2020 (*n* = 75)		Total (*n* = 352)	
	*N*	(%)	*N*	(%)	*N*	(%)	*N*	(%)
Referrals with diagnosed or suspected ASD	47	(27.0)	37	(35.0)	27	(36.0)	111	(31.5)
Total admissions	45	(26.3)	33	(31.1)	28	(37.3)	106	(30.1)
Admissions with diagnosed or suspected ASD	10	(22.0)	10	(30.0)	10	(36.0)	30	(8.5)

Over the three-year period, *n* = 30 YP with diagnosed (*n* = 10) or suspected (*n* = 20) ASD were admitted to the unit, which constituted 8.5% of total referrals and 28.3% of total admissions. Chi-square analyses showed that YP with suspected or confirmed ASD were not more likely to be admitted than those without ASD, χ2 = 0.73, *p* = .392.

### Demographics

The characteristics of YP with diagnosed or suspected ASD (*n* = 111) referred to the service, including gender, admission status and range of co-occurring conditions are presented in Table 2. The average age was 15.3 years old (SD = 1.46). Of the 111 young people with diagnosed or suspected ASD, more males (55.0%) than females (45.0%) were referred to the unit (Table 2). Additionally, a higher percentage of males (50.9%) presented at time of referral with a formal diagnosis of ASD versus a suspected ASD diagnosis than females (38.0%). While the sample of young people with autism also included 6 individuals who were assigned female at birth but identified as male (FTM), these were included within the male sample to facilitate statistical analysis. Other than rates of gender incongruence, there were no statistically significant differences across the variables (Table 3).

**Table 2.** Characteristics of Referred Young People With Diagnosed or Suspected ASD^a^ (*n* = 111).

	*N*	%
Male	61	55.0
Admitted to unit	30	27.0
Depressive disorders	55	49.5
Anxiety disorders	55	49.5
Suicidality	31	27.9
Schizophrenia spectrum and other psychotic disorders	21	18.9
Deliberate self-harm	21	18.9
Obsessive-compulsive and related disorders	16	14.4
Trauma- and stressor-related disorders	15	13.5
Feeding and eating disorders	14	12.6
ADHD	12	10.8
Personality development difficulties	12	10.8
Bipolar and related disorders	9	8.1
Disruptive, impulse control and conduct disorders	8	7.2
Gender dysphoria	7	6.3
Substance-related and addictive disorders	2	1.8

^a^Refers to diagnosed or suspected ASD as stated in referral form.

**Table 3.** Characteristics of young people referred With diagnosed or suspected ASD by gender (*n* = 111).

	Male		Female		FTM	
	(*n* = 55)		(*n* = 50)		(*n* = 6)	
	*N*	(%)	*N*	(%)	*N*	(%)
ASD diagnosis	28	(50.9)	19	(38.0)	2	(33.3)
Suspected ASD	27	(49.1)	31	(62.0)	4	(66.7)
Admitted to unit	13	(23.6)	14	(28.0)	3	(50.0)
Depressive disorders	32	(58.2)	21	(42.0)	2	(33.3)
Anxiety disorders	26	(47.3)	26	(52.0)	3	(50.0)
Suicidality	16	(29.1)	12	(24.0)	3	(50.0)
Schizophrenia spectrum and other psychotic disorders	12	(21.8)	8	(16.0)	1	(16.7)
Deliberate self-harm	8	(14.5)	11	(22.0)	2	(33.3)
Obsessive-compulsive and related disorders	13	(23.6)	3	(6.0)	0	(0.0)
Trauma- and stressor-related disorders	8	(14.5)	6	(12.0)	1	(16.7)
Feeding and eating disorders	1	(1.8)	13	(26.0)	0	(0.0)
ADHD	8	(14.5)	4	(8.0)	0	(0.0)
Bipolar and related disorders	2	(3.6)	7	(14.0)	0	(0.0)
Disruptive, impulse control and conduct disorders	4	(7.3)	4	(8.0)	0	(0.0)
Gender dysphoria	0	(0.0)	3	(6.0)	4	(66.7)
Substance-related and addictive disorders	2	(3.6)	0	(0.0)	0	(0.0)
Personality development difficulties	2	(3.6)	9	(18.0)	1	(16.7)

YP with diagnosed or suspected ASD who were referred to the unit presented with a wide range of co-occurring conditions. Overall, 18.9% (*n* = 21) presented with one co-occurring condition only, 37.0% (*n* = 41) presented with two, 33.3% (*n* = 37) presented with three, 8.1% (*n* = (9) presented with four, 0.9% (*n* = (1) presented with five and 1.8% (*n* = (2) presented with six co-occurring conditions. Tables 2 and 3 show the co-occurring conditions. Depression and anxiety disorders were the most common mental health difficulties present alongside diagnosed or suspected ASD. Suicidality was also a common presentation among those referred with diagnosed or suspected ASD, followed by DSH, schizophrenia spectrum and other psychotic disorders, obsessive-compulsive and related disorders, trauma and stressor-related disorders, and feeding and eating disorders.

Of the 30 YP with diagnosed or suspected ASD who were admitted to the unit, 20% (*n* = 6) were engaged with an ASD specialist service at the time of admission.

This figure rose to 50% (*n* = 5), when only the 10 YP with a formal diagnosis of ASD were considered.

Chi square tests for independence were computed to determine whether there were significant associations between status of ASD diagnosis (formal diagnosis or suspected), gender (male or female), admission status (admitted or not admitted) and co-occurring conditions (Table 4).

**Table 4.** Characteristics of young people referred With diagnosed or suspected ASD (*n* = 111) and associations Between status of autism diagnosis and co-occurring conditions.

	ASD diagnosis (*n* = 49)		Suspected ASD (*n* = 62)		X^2^	df	*p*-value	Cramer’s V
	*n*	(%)	*n*	(%)				
Gender								
Male	30	(61.2)	31	(50.0)	1.39	1	0.238	0.112
Referral status								
Admitted	10	(20.4)	20	(32.3)	1.95	1	0.163	0.132
Co-occurring condition								
Depressive disorders	20	(40.8)	35	(56.5)	2.67	1	0.102	0.155
Anxiety disorders	23	(46.9)	32	(51.6)	0.24	1	0.625	0.046
Suicidality	15	(30.6)	16	(25.8)	0.31	1	0.575	0.053
Schizophrenia spectrum and other psychotic disorders	7	(14.3)	14	(22.6)	1.23	1	0.268	0.105
Deliberate self-harm	11	(22.4)	10	(16.1)	0.71	1	0.399	0.080
Obsessive-compulsive and related disorders	10	(20.4)	6	(9.7)	2.56	1	0.110	0.152
Trauma- and stressor-related disorders	9	(18.4)	6	(9.7)	1.89	1	0.169	0.131
Feeding and eating disorders	3	(6.1)	11	(17.7)	3.35	1	0.067	0.174
ADHD	9	(18.4)	3	(4.8)	5.20	1	0.023*	0.216
Personality development difficulties	6	(12.2)	6	(9.7)	.19	1	0.665^a^	.041
Bipolar and related disorders	4	(8.2)	5	(8.1)	-	-	1.000^a^	-
Disruptive, impulse control and conduct disorders	8	(16.3)	0	(0.0)	-	-	0.001*^a^	-
Gender dysphoria	1	(2.0)	6	(9.7)	-	-	0.131^a^	-
Substance-related and addictive disorders	0	(0.0)	2	(3.2)	-	-	0.502^a^	-

Note. ADHD = attention deficit hyperactivity disorder; df = degrees of freedom.

*Note.* Tests of association were not conducted where 50% of cell counts were less than 5.

^a^Reflects result of Fisher’s Exact Test as the assumption of expected cell count >5 was violated for Chi Square test.

*p ≤ .05.

Results showed a statistically significant association between status of ASD diagnosis and ADHD (Table 4). YP with a formal diagnosis of ASD were more likely to also have an ADHD diagnosis (18.4%–4.8%) and this association was of small effect, Cramer’s V = 0.216, *p* = .023. A Fisher’s Exact test was conducted between status of ASD diagnosis and disruptive, impulse control and conduct disorders. Results indicated a statistically significant association (*p* = .001), where YP with a formal diagnosis of ASD were more likely to also have a diagnosis of a disruptive, impulse control and conduct disorder (16.3%–0%). No other co-occurring conditions differed by status of ASD diagnosis.

### Gender differences

Chi square tests for independence results by gender are presented in Table 5. Results indicated a statistically significant association between gender and obsessive, compulsive and related disorders. YP with diagnosed or suspected ASD who identified as male were significantly more likely to present with obsessive, compulsive and related disorders (21.3%–6.0%). This association was of small effect, Cramer’s *V* = 0.217. Additionally, results showed that YP with suspected or diagnosed ASD who identified as female were significantly more likely to present with feeding and eating disorders (26.0%–1.6%). This association was of medium effect, Cramer’s V = 0.365, *p* < .001. Females with diagnosed or suspected ASD were also more likely than males to present with personality development difficulties (18%–4.9%), which was a small effect; Cramer’s *V* = 0.21, *p* = .027. No other co-occurring conditions differed by gender.

**Table 5.** Characteristics of young people referred With ASD by gender (*n* = 111).

	Male (*n* = 61)		Female (*n* = 50)					
	*n*	(%)	*n*	(%)	X^2^	df	*p*-value	Cramer’s V
Status of autism diagnosis								
Formally diagnosed	30	(49.2)	19	(38.0)	1.39	1	0.238	0.112
Referral status								
Admitted	16	(26.2)	14	(28.0)	0.04	1	0.834	0.020
Co-occurring condition								
Depressive disorders	34	(55.7)	21	(42.0)	2.07	1	0.150	0.137
Anxiety disorders	29	(47.5)	26	(52.0)	0.22	1	0.640	0.044
Suicidality	19	(31.1)	12	(24.0)	0.70	1	0.404	0.079
Schizophrenia spectrum and other psychotic disorders	13	(21.3)	8	(16.0)	0.51	1	0.477	0.067
Deliberate self-harm	10	(16.4)	11	(22.0)	0.56	1	0.453	0.071
Obsessive-compulsive and related disorders	13	(21.3)	3	(6.0)	5.22	1	0.022*	0.217
Trauma- and stressor-related disorders	9	(14.8)	6	(12.0)	0.15	1	0.703	0.036
Feeding and eating disorders	1	(1.6)	13	(26.0)	14.80	1	<0.001*	0.365
ADHD	8	(13.1)	4	(8.0)	0.75	1	0.388	0.082
Bipolar and related disorders	2	(3.3)	7	(14.0)	4.24	1	0.076^a^	.195
Personality development difficulties	3	(4.9)	9	(18.0)	4.88	1	0.034a*	.210
Disruptive, impulse control and conduct disorders	4	(6.6)	4	(8.0)	-	-	1.000^a^	-
Gender dysphoria	4	(6.6)	3	(6.0)	-	-	1.000^a^	-
Substance-related and addictive disorders	2	(3.3)	0	(0.0)	-	-	0.500^a^	-

*Note.* ADHD = attention deficit hyperactivity disorder; df = degrees of freedom.

*Note.* Tests of association were not conducted where 50% of cell counts were less than 5.

^a^Reflects result of Fisher’s Exact Test as the assumption of expected cell count >5 was violated for Chi Square test.

*p ≤ .05.

### Admission status

Finally, a chi-square test of independence was conducted between admission status (admitted or not admitted) and reported suicidality (yes or no) to explore whether suicidality influenced admission to the unit. There was a statistically significant association between admission status and suicidality, χ2 (1) = 9.950, *p* = .002. YP with diagnosed or suspected ASD who reported suicidality were significantly more likely to be admitted to the unit than those who were not suicidal. This association was of medium effect, Cramer’s *V* = 0.299, *p* = .002. There was no association with deliberate self-harm and admission status.

## Discussion

To our knowledge, this is the first study to date to examine the clinical and demographic profile of YP with diagnosed or suspected ASD referred to CAMHS inpatient services in Ireland. There appears to be a paucity of literature pertaining to the prevalence of ASD in CAMHS services therefore, comparative research is limited for the discussion below. This gap in research appears to be particularly pronounced in an Irish context.

The results of our study demonstrated that YP with diagnosed or suspected ASD constituted 31.5% of total referrals and 28.3% of total admissions to the inpatient unit over a three-year period. Of 111 referrals indicating ASD, 44.1% had a formal diagnosis while 55.9% had suspected ASD only. This result is significant as it is the first examination of the prevalence of ASD (both confirmed and suspected) in referrals to a generic inpatient CAMHS unit in an Irish context. The novel examination arose from the necessity to highlight the clinical implications associated with triaging and care-planning for YP without a formal diagnosis of ASD. There are myriad factors which may contribute to a YP being referred without a formal diagnosis or the differential diagnosis being identified by the referral agent in the first instance. Lengthy waiting lists, limited access to early intervention, Primary Care and CDNTs may stymie early identification of ASD needs in childhood. A burgeoning yet limited body of evidence indicates that children and YP with ASD are at risk of developing mental health difficulties due to the distress which can arise from not having their ASD specific needs identified or met (Au-Yeung et al., 2019; Orji & Sharkey, 2021; Maguire, Glynn, McGrath, & Byrne, 2023). The high referral rates for YP with suspected or diagnosed ASD also shows the increased risk for youth with autism of being referred for inpatient care. Nevertheless, despite the proportionally high referral rate, those with a diagnosis of ASD were not more likely than youth without autism to be admitted to the unit. This demonstrates the fact that the inpatient CAMHS service may not be most appropriate to their needs, but it raises the important question of where these YP can access support, as highlighted by the Mental Health Commission (2018).

YP with diagnosed or suspected ASD in our study presented with a wide range of co-occurring psychiatric conditions and multiple comorbidities in some cases. This corresponds with available literature. Simonoff et al. (2008) found that 70% of young people with ASD in a UK sample met criteria for one psychiatric diagnosis while 41% met criteria for two or more. Depression and anxiety disorders were the most common co-occurring conditions present in our study, with almost half of the young people referred to the unit with diagnosed or suspected ASD presenting with these difficulties (49.5%). Rosenberg et al. (2011) identified that after ADHD, anxiety and depression are the most common co-morbidities for YP with ASD. This is unsurprising, as the presence of mood disorders in youth with autism has been found to be highly correlated with inpatient hospitalisation (Righi et al., 2018; Hossain et al., 2020). Similar to the findings of Maguire et al. (2023) albeit in a younger population, suicidality was a common presentation among the YP with diagnosed or suspected ASD who were referred (27.9%) and those presenting with suicidality were significantly more likely to be admitted to the inpatient unit in question. Other common presenting problems were deliberate self-harm (18.9%), schizophrenia spectrum and other psychotic disorders (18.9%), obsessive-compulsive and related disorders (14.4%), trauma and stressor-related disorders (13.5%), and feeding and eating disorders (12.6%).

We were also able to explore gender differences in presentations amongst YP with autism. Our results showed that male YP with diagnosed or suspected ASD were more likely to also have a diagnosis of OCD. This is consistent with previous work by Martin et al. (2020) which highlighted that young people with OCD were significantly more likely to be male (64%). In a UK community CAMHS setting, Sethlare and Rafi (2022), identified obsessional symptoms which were more common in boys with ASD (63.3%) as compared to girls (27.3%). Our findings highlighted that female YP with ASD were significantly more likely to present with feeding and eating disorders. Females with autism may present more often with a diagnosis of an eating disorder due to the similarities in cognitive and socioemotional functioning (Oldershaw et al., 2011); Westwood & Tchanturia, 2017). A recent qualitative study highlighted that women with ASD experienced their anorexia nervosa as closely linked with their ASD, intertwined with their need for control and predictability, rigid thinking styles, sensory sensitivities and their sense of self and identity (Brede et al., 2020). Similarly, females with suspected or confirmed ASD in this study were more likely to be referred with personality development difficulties. This corresponds with recent literature which demonstrates that women with autism are often identified as having a diagnosis of personality disorder before receiving an autism diagnosis (Fusar-Poli et al., 2022).

Hossain et al. (2020) reports that in co-morbidities among individuals with ASD globally, ADHD prevalence rates ranged from 25.7% to 65.0%. Our results showed lower overall rates of co-occurring ADHD at 10.8%, although young people with a formal ASD diagnosis were significantly more likely to also have a diagnosis of ADHD. Comparably, the population with ASD identified as having ADHD was 14.7% by Leader et al. (2022) and 6% in Maguire et al. (2023), although these studies encompassed a younger population than the present study.

We explored whether there were any particular factors associated with admission to the unit among YP with ASD. Results showed that young people with diagnosed or suspected ASD who reported suicidality were significantly more likely to be admitted to the unit. This finding represents a crucial data point, necessitating further service level planning for the risk characteristic of this population. Previous research shows that YP with ASD are 28 times more likely to experience suicidal ideation or attempt suicide than neurotypical YP (Cusack, Shaw, Spiers, & Sterry, 2016). Within an Irish setting, comparisons could be drawn with the results of Maguire et al. (2023), where the authors identified that half of children with ASD in their study presented with suicidal thoughts and behaviours indicative of significant psychological stressors in this population.

Finally, only six of the YP with suspected or confirmed ASD who were admitted to the unit (*n* = 30) were concurrently clients of an autism or disability service at the time of admission. This means that there was no immediate access for inpatient CAMHS clinicians to consultation or collaborative working with relevant services for the vast majority of these YP. In order to ensure comprehensive care and access to the services to which these YP are entitled, responsibility for interagency advocacy and care co-ordination falls to the inpatient team in the first instance, demanding significant clinical and time resources.

The findings of this study have important clinical and resource implications. For inpatient clinicians triaging referrals, the suspicion of an ASD as a differential diagnosis presents significant considerations for clinical practice. Such cases often require additional intervention considerations and care coordination, including onward referral for assessment for a YP’s potential ASD specific needs. This is particularly involved in instances where a young person with autism is not actively engaged in specialist services. The sizeable number of referrals with a confirmed or suspected diagnosis of ASD highlights the prevalence of this issue in clinical practice and a significant resourcing need pertaining to staff training, autism-informed practice and appropriate infrastructure in the unit environment to facilitate the needs of this population. As the current study pertains to a single publicly funded unit, generalisability is limited. However, in the absence of other comparable data in an Irish context, one can surmise that this might reflect referral trends nationally, which may have important implications for service configuration. For example, it may mean that greater autism-specific resourcing is required within generic inpatient CAMHS services. It also highlights the necessity for a joint approach between disability and mental health services for YP with co-occurring autism and complex mental health needs, as recommended by the HSE (HSE, 2017).

### Limitations

This study should be interpreted in light of its limitations. The included sample is from one CAMHS inpatient unit in Ireland, which limits generalisability. Future investigations comparing the results of this study with data from the remaining three inpatient units in Ireland may be beneficial. A further limitation exists in that the timespan examined in the present study incorporated two years in which a reduction in referrals to the unit were observed due to the COVID-19 pandemic. The results may reflect an under-representation of the prevalence of young people with ASD requiring CAMHS inpatient support. Over the period examined, the year 2020 saw the fewest referrals to the inpatient unit, which is consistent with findings by McNicholas et al. (2021) who observed a significant drop in referrals to specialist CAMHS during the 2020 COVID-19 pandemic, compared with 2018 and 2019. While we explored whether an ASD diagnosis was formally given or queried, the method of diagnosis (e.g., best practice guidelines or ‘gold standard’; Kaufman [2022]) was not readily available on the referral forms and was therefore not collected. Additionally, it is important to acknowledge that psychiatric co-morbidities identified or queried on referral forms may not represent the true nature of a young person’s difficulties, which may only be reliably determined over time. Furthermore, we did not collect data on the number of individuals referred to the service, rather than number of referrals made. While readmission rates were very rare in the service, it is important for future research to take account of re-referral and re-admission rates in order to gain a clear picture of the needs of this population.

In summary, this novel study found high rates of suspected or confirmed autism among referrals of YP to an inpatient CAMHS service in Ireland, as well as detailing the most common presenting difficulties in this population and the lack of autism-specific services for YP. The results have important implications for clinical practice, as they highlight the need for inpatient CAMHS services to be able to access timely, collaborative interagency working with disability and primary care services for YP with autism and co-occurring severe and complex mental health difficulties. The study also demonstrates the importance of joint working models between disability and mental health services so that YP with ASD or queried ASD can receive the full range of clinical input needed.
